# Elovl5 is required for proper action potential conduction along peripheral myelinated fibers

**DOI:** 10.1002/glia.24048

**Published:** 2021-06-17

**Authors:** Eriola Hoxha, Ilaria Balbo, Roberta Parolisi, Matteo Audano, Francesca Montarolo, Francesco Ravera, Michela Guglielmotto, Luisa Muratori, Stefania Raimondo, Eleonora DiGregorio, Annalisa Buffo, Alfredo Brusco, Barbara Borroni, Nico Mitro, Donatella Caruso, Filippo Tempia

**Affiliations:** ^1^ Neuroscience Institute Cavalieri Ottolenghi (NICO) Orbassano Italy; ^2^ Department of Neuroscience University of Torino Torino Italy; ^3^ Department. of Pharmacological and Biomolecular Sciences Università degli Studi di Milano Milan Italy; ^4^ Department of Clinical and Biological Sciences University of Torino Torino Italy; ^5^ Medical Genetics Unit, Città della Salute e della Scienza Hospital and Dept. of Medical Sciences University of Torino Torino Italy; ^6^ Centre for Neurodegenerative Disorders, Department of Clinical and Experimental Sciences University of Brescia Brescia Italy; ^7^ National Neuroscience Institute Torino Italy

**Keywords:** action potential, axon, Elovl5, myelin, polyunsaturated fatty acids

## Abstract

Elovl5 elongates fatty acids with 18 carbon atoms and in cooperation with other enzymes guarantees the normal levels of very long‐chain fatty acids, which are necessary for a proper membrane structure. Action potential conduction along myelinated axons depends on structural integrity of myelin, which is maintained by a correct amount of fatty acids and a proper interaction between fatty acids and myelin proteins. We hypothesized that in *Elovl5* ^*−/−*^ mice, the lack of elongation of Elovl5 substrates might cause alterations of myelin structure. The analysis of myelin ultrastructure showed an enlarged periodicity with reduced G‐ratio across all axonal diameters. We hypothesized that the structural alteration of myelin might affect the conduction of action potentials. The sciatic nerve conduction velocity was significantly reduced without change in the amplitude of the nerve compound potential, suggesting a myelin defect without a concomitant axonal degeneration. Since Elovl5 is important in attaining normal amounts of polyunsaturated fatty acids, which are the principal component of myelin, we performed a lipidomic analysis of peripheral nerves of Elovl5‐deficient mice. The results revealed an unbalance, with reduction of fatty acids longer than 18 carbon atoms relative to shorter ones. In addition, the ratio of saturated to unsaturated fatty acids was strongly increased. These findings point out the essential role of Elovl5 in the peripheral nervous system in supporting the normal structure of myelin, which is the key element for a proper conduction of electrical signals along myelinated nerves.

## MAIN POINTS

- Consequences on peripheral nerves of Elovl5 deletion:
  - Unbalance between saturated versus unsaturated fatty acids in membrane phospholipids.
  - Loss of myelin compactness
  - Slower action potential conduction

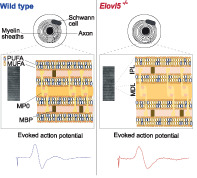

## 1 INTRODUCTION

Myelin sheaths wrapped around axons enable saltatory propagation of action potentials, thereby increasing their velocity (Suminaite et al., 2019). Myelin, constituted by multiple layers of lipid‐rich membranes, provides electrical insulation preventing leakage of current by increasing the resistance between the axonal cytoplasm and the interstitial fluid (Bakiri et al., 2011). This effect increases the space constant, promoting faster transfer of action potentials from one node of Ranvier to the next. At the same time, myelin reduces membrane capacitance at the internode, thereby reducing the time constant, so that electrical charging becomes faster, speeding up action potential conduction. These properties are strictly dependent on the compactness of myelin layers, which assures that internodes are almost entirely formed by lipid membranes with minimal cytoplasmic content (Simons, 2016).

Lipids are the main component of myelin, constituting about 80% of its dry mass (O'Brien & Sampson, 1965; Quarles et al., 2005). In myelin, the main lipid classes are phospholipids (glycerophosphatides), sphingolipids and cholesterol (Norton & Poduslo, 1973; O'Brien & Sampson, 1965). The structural integrity of myelin depends on the interaction between lipids and membrane proteins (Bradl, 1999; Min et al., 2009; Ohler et al., 2004). Even subtle alterations in lipid or protein composition can disrupt the normal myelin structure and function (Maganti et al., 2019). Notably, mice lacking the lipogenic transcription factor sterol regulatory element‐binding factor‐1c (Srebf1c) have blunted peripheral nerve fatty acid synthesis that results in myelin alterations leading to development of peripheral neuropathy (Cermenati et al., 2015). The chains of fatty acids of phospholipids and sphingolipids are a main determinant of their structural and physical properties, which are critical for myelin function. The length and the number of unsaturated bonds are particularly important. Fatty acids are synthesized in several tissues, including the brain, or are introduced with the diet. Polyunsaturated fatty acids (PUFA) cannot be synthesized by mammals, so that they must be provided by nutrition. The essential PUFAs, from which the others can be derived, are linoleic acid and alpha‐linolenic acid, which have 18 carbon atoms. Starting from such essential PUFAs, a number of very long‐chain fatty acid elongases (ELOVL) and desaturases can produce a variety of downstream molecules with 20 carbon atoms or more, which possess either structural or signaling functions (Guillou et al., 2010; Serhan et al., 2014). Seven ELOVL enzymes are known, with ELOVL1, ELOVL3, ELOVL6, and ELOVL7 preferring saturated or monounsaturated fatty acids, while ELOVL2, ELOVL4, and ELOVL5 being more selective for PUFAs. More specifically, the step of elongation from 18 to 20 carbon atoms mainly relies on ELOVL5, as liver microsomes lacking this enzyme are almost unable to elongate PUFAs with 18 carbon atoms (Moon et al., 2009). Based on these premises, the role fatty acid elongases and specifically that of ELOVL5 on alterations of myelin lipid composition remains still unknown.

Mice with a targeted deletion of *Elovl5* (*Elovl5* ^*−/−*^) show neurologic deficits (Hoxha et al., 2017), in line with an essential role of downstream PUFAs in the nervous system. We hypothesized that the lack of Elovl5, by causing a specific modification in the fatty acid composition of phospholipids, might result in structural alterations of myelin. The ultrastructural analysis of myelin in the peripheral nerve of Elovl5^−/−^ mice revealed an altered periodicity of myelin layers, in agreement with a role of long chain fatty acids in determining the optimal thickness of the lamellae. Such structural alteration is accompanied by a strong reduction of long‐chain PUFA, while saturated fatty acids are increased. The functional consequence of the structural and molecular alterations consists of a decreased action potential velocity.

## 2 MATERIALS AND METHODS

### 2.1 Animals

*Elovl5* knockout mice (*Elovl5* ^*−/−*^), of the C57BL/6 genetic background, have been kindly provided by Dr. Moon and Dr. Horton of the UT Southwestern Medical Center (Moon et al., 2009). Experimental animals and wild type controls were obtained by mating heterozygous *Elovl5* ^+/−^ mice, with the expected Mendelian frequency of ¼ *Elovl5* ^−/−^ and ¼ *Elovl5* ^+/+^. Heterozygos littermates were discarded. Animals were kept on a natural diet without animal derivatives (Teklad Global 18% Protein Rodent Diet, Harlan Laboratories) and both female and male mice (12 months old) were used for all the experimental paradigms. All experimental procedures have been approved by the Ethical Committee of the University of Torino and authorized by the Italian Ministry of Health (authorization number: 161/2016‐PR).

### 2.2 High resolution light microscopy and transmission electron microscopy

High resolution light and transmission electron microscopy were carried out as reported in (Mancini et al., 2019). Mice were anesthetized by intraperitoneal injection with ketamine (100 mg/kg body weight) and xylazine (10 mg/kg body weight). For each animal the sciatic nerve was exposed, and a nerve segment was removed. Samples were first fixed in 2.5% glutaraldehyde in 0.1 M phosphate buffer (pH 7.4) for at least 4 h at 4°C and then were postfixed with 2% osmium tetroxide for 2 h and dehydrated in ethanol from 30% to 100% (5 min each passage). After two passages of 7 min in propylene oxide and 1 h in a 1:1 mixture of propylene oxide and Glauerts' mixture of resins, samples were embedded in Glauerts' mixture of resins (made of equal parts of Araldite M and the Araldite Harter, HY 964, Sigma Aldrich). In the resin mixture, 0.5% of the plasticizer dibutyl phthalate (Sigma Aldrich) was added. For the final step, 2% of accelerator 964 was added to the resin in order to promote the polymerization of the embedding mixture, at 60°C. Transverse semithin sections (2.5 μm thick) were obtained using an ultramicrotome (Ultracut UCT, Leica, Wetzlar, Germany), and stained with 1% toluidine blue and 2% borate in distilled water for high resolution light microscopic examination and design‐based stereology. A DM4000B microscope equipped with a DFC320 digital camera was used for section analysis. Quantification of total number of myelinated nerve fibers was performed on toluidine blue‐stained semi‐thin sections. At this purpose, the two‐dimensional disector method (Geuna et al., 2000) together with a systematic random sampling scheme was applied: 12–16 sampling fields were randomly selected and, in each field, the two‐dimensional disector procedure was carried out. The total cross‐sectional area of the whole nerve was also measured and used to calculate the total number of myelinated fibers (Geuna et al., 2000; Mancini et al., 2019). The G‐ratio (inner perimeter/outer perimeter), the axon diameter (as frequency distribution), the myelin sheath thickness and myelin periodicity were analyzed in ultrathin (70–100 nm) sections using a transmission electron microscope (JEOL, JEM‐1010, Tokyo, Japan) equipped with a Mega‐View‐III digital camera and a Soft‐Imaging‐System (SIS, Münster, Germany) for computerized acquisition of the images. The analysis was performed using ImageJ software (https://imagej.net/Fiji, RRID: SCR_003070). The quantifications of G‐ratio, axon diameter and myelin sheath thickness were performed on at least 125 axons/animal and on 3–5 mice per genotype.

### 2.3 Immunohistochemistry and confocal microscopy

*Elovl5*^*−/−*^(n = 3) and wild type (n = 3) littermates were anesthetized with isoflurane (Isoflurane‐Vet, Merial, Milan, Italy) and decapitated. Sciatic nerves were then exposed, separated from the surrounding muscular tissue and dissected. Samples were fixed in cold 4% paraformaldehyde for 30 min subsequently transferred in cold 0.1 M phosphate buffered saline (1x PBS) pH 7.2–7.4, and stored overnight at 4°C. The following day, nerves were embedded in optimal cutting temperature compound (OCT), serially cut by a cryostat (Leica CM1900, Leica Microsystem, Milan, Italy) in 50 μm‐thick longitudinal slices and stored at 4°C until usage. Floating slices were incubated with blocking solution (0.5% Triton X100, 5% Normal Goat Serum [NGS], in 1x PBS) for 1 h at room temperature.

Immunofluorescence labeling was performed incubating slices with primary antibody mouse monoclonal anti‐Caspr (1:500, kindly gifted by Professor Elior Peles) diluted in a 0.1% triton X‐100 solution and 5% NGS one night at 4°C. The following day, sections were washed three times in 1x PBS (15 min) and then incubated with goat anti‐mouse IgG1 secondary antibody Cy5 (1:300, kindly gifted by Professor Elior Peles) and DAPI (1:1000, Fluka, Saint Louis, United States) diluted in a 0.1% triton X‐100 solution and 5% NGS, 45 min at room temperature. Finally, slices were washed three times (15 min) in 1x PBS, mounted and, when dry, glass coverslip was applied using Mowiol (Calbiochem, 308 LaJolla, CA, United States).

The nodal gap and paranode length analysis were performed on confocal images acquired using 63X oil objectives with a Leica TCS SP5 (Leica Microsystems, Milan, Italy) confocal microscope. Images (1024 × 1024 pixel, 0.50 μm thick optical sections) were analyzed using ImageJ software (1.52 t version). At least three slices/animal were analyzed.

### 2.4 Caudal nerve conduction velocity

Mice were anesthetized via intraperitoneal injection with ketamine (100 mg/kg body weight) and xylazine (10 mg/kg body weight). Stainless steel subdermal needle electrodes (Technomed, medical accessories) were used to deliver supramaximal stimulation with 0.05 ms impulses using an isolated stimulator (A‐M System 2100, Sequim, WA, USA). Low frequency filters were set to 300 Hz and high frequency filters were set to 10 kHz.

The nerve conduction velocity (NCV) in the tail nerve was assessed by placing needle electrodes in the tail, with the positive recording electrode at a few centimeters from the base of the tail. In proximal to distal direction the distances from the first electrode were: negative recording electrode 0.5 cm, ground 1.5, negative stimulation electrode 4 cm, positive stimulation electrode 4.5 cm. The latency of the potentials recorded after nerve stimulation was measured and the NCV was calculated accordingly. All neurophysiological determinations were performed under standard conditions and external body temperature was maintained at 34°C with a heating lamp.

### 2.5 Protein analysis

Sciatic nerves from wild type (n = 8) and *Elovl5* ^*−/−*^ (n = 4) mice were resuspended in 20% (w/v) RIPA buffer (25 mM Tris–HCl pH 7.4, 150 mM NaCl, 1 mM EGTA, 1 mM EDTA, 1 mM dithiothreitol, 0.5 mM PMSF, 10 μg/ml Aprotinine, 10 μg/ml Leupeptine, 2 mM sodium orthovanadate), and homogenized with a tissue lyser (Russo et al., 2018). The lysates were centrifuged at 10,000 g for 20 min at 4°C and the supernatant was collected and stored at −80°C until use. Twenty micrograms of proteins were separated by using a 4–12% Bis‐Tris precast gel (Life Technologies) and transferred onto nitrocellulose membrane (GE‐Healthcare). Membranes were than blocked with 50 g/L (5%) nonfat dry milk (Bio‐Rad) in 50 mM Tris–HCl pH 7.4, containing 200 mM NaCl and 0.5 mM Tween‐20 and then incubated overnight at 4°C with primary antibodies. The following primary antibodies were used: MBP (1:1000, Covance, Cat# SMI‐99P‐500, RRID: AB_10120130), CNP‐ase (1:500, Millipore, Cat# MAB326R, RRID: AB_94780), MPZ (1:3000, GeneTex Cat# GTX134070, RRID: AB_2876362), β‐Actin (1:16000, Abcam, Cat# ab8226, RRID: AB_306371). HRP‐conjugated goat anti‐mouse (1:5000, Bio‐Rad, Cat# 170–6516, RRID: AB_11125547) and goat anti‐rabbit (1:5000, Bio‐Rad, 170–6515, RRID: AB_11125142) immunoglobulins, in Tris‐buffered saline Tween containing 20 g/L non‐fat dry milk, was used for detection with Luminata Forte Western substrate (WBLUF0100, Millipore). Densitometric values were normalized to β‐Actin. Images were acquired by Chemidoc (Bio‐Rad) and quantified by ImageLab software (RRID: SCR_014210, Bio‐Rad).

### 2.6 Liquid chromatography–tandem mass spectrometry analysis

The levels of total phospholipids were evaluated by means Liquid Chromatography–tandem mass spectrometry (LC)‐MS/MS according to published protocol (Cermenati et al., 2015) with some modifications described below. Briefly, internal standards (13C‐all labeled linoleic acid and 13C‐all labeled palmitic acid) were added to samples (10 mg for tissues), and lipid extraction was performed using 1 ml of methanol (MeOH)/Acetonitrile (1/1; v/v). Phospholipids analysis: methanolic extracts were analyzed by liquid chromatography tandem mass spectrometry (LC–MS/MS) using XTerra Reverse Phase C18 column (3.5 μm 4.6 x 100 mm, Waters) and as isocratic mobile phase MeOH with 0.1% formic acid with 274 multiples reaction monitoring (MRM) transitions for positive ion mode in 5 min total run for each sample. LC‐ESI‐MS/MS for negative ion mode was conducted with a cyano‐phase LUNA column (50 mm x 4.6 mm, 5 μm; Phenomenex) and as isocratic mobile phase 5 mM ammonium acetate pH 7 in MeOH with 50 MRM transitions in 5 min total run for each sample. The identity of the different phospholipid families was confirmed using pure standards, namely one for each family. An ESI source connected with an API 4000 triple quadrupole instrument (AB Sciex, USA) was used. MultiQuantTM software version was used for data analysis and peak review of chromatograms. Changes between detected phospholipid families were calculated as percent of single phospholipid species normalized to total phospholipid analyzed. Data points have been graphically displayed by the “ggplot2” package of “R” programming language.

### 2.7 Statistical analyses

The Shapiro–Wilk or Kolmogorov–Smirnov test was first applied to test for a normal distribution of the data. When data were normally distributed, two‐tailed unpaired Student's *t*‐test was used. Alternatively, for data not normally distributed, Mann–Whitney *U*‐test has been used. Statistical tests were performed by means of SPSS software (SPSS Inc., Chicago, IL, USA, RRID: SCR_002865). *p*‐values <.05 were accepted as significant.

## 3 RESULTS

### 3.1 Structural alterations of sciatic nerve of *Elovl5* ^*−/−*^ mice

**FIGURE 1:**
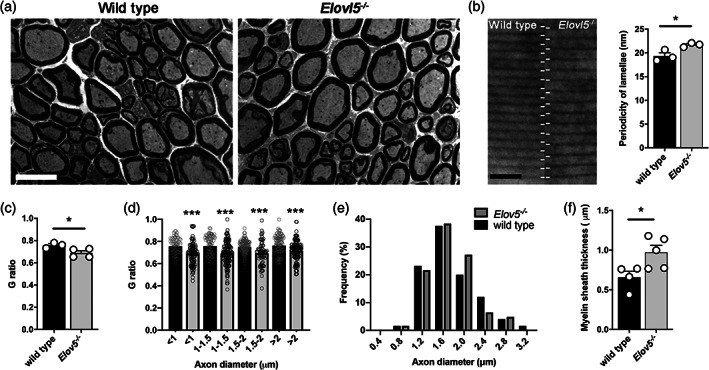
Structural alterations of sciatic nerve of *Elovl5* ^−/−^ mice. (a) Representative images of fibers in wild type and in *Elov5* ^*−/−*^ sciatic nerves (scale Bar = 10 μm). (b) Ultra structural representation of myelin periodicity (scale Bar = 40 nm) and of the distance between consecutive major dense lines (*Elov5* ^*−/−*^ n = 3 mice vs. wild type n = 3 mice). (c) bar graph representing the G‐ratio (inner perimeter/outer perimeter) of sciatic nerve myelinated fibers (*Elov5* ^*−/−*^ n = 4 mice vs. wild type n = 3 mice). (d) Graph representing the relative frequency of axonal diameters (μm) (*Elov5* ^*−/−*^ n = 4 vs. wild type n = 3). (e) Bar graph representing myelin sheath thickness (mm) of sciatic nerve myelinated fibers (*Elov5* ^*−/−*^ n = 5 vs wild type n = 4). (f) G‐ratio quantification of fibers accounting for axon diameter (*Elov5* ^*−/−*^ n = 4 vs. wild type n = 3). Data are expressed as mean ± SEM and *p*‐values are determined by the appropriate statistical test. *p < .05; ***p < .001

Stereological analysis of sciatic nerve fibers showed no significant difference between wild type (n = 4 mice) and *Elovl5* ^*−/−*^ mice (n = 5) in the total number of myelinated fibers (wild type: 3441 ± 213.1; *Elovl5* ^*−/−*^mice: 3104 ± 217.4; Unpaired Student's *t*‐test *t*_[7]_ = 1.09, *p* > .05). Myelin ultrastructure analysis revealed that sciatic nerve fibers of *Elovl5* ^*−/−*^ mice displayed a lower G‐ratio compared to control littermates (wild type: 0.76 ± 0.01; *Elovl5* ^*−/−*^ mice: 0.69 ± 0.02; Student's *t*‐test *t*_[5]_ = 2.60, *p* < .05, Figure 1(a,c)). The G‐ratio was smaller for fibers of any caliber (Figure 1(d), *p* < .001, Mann–Whitney *U*‐test). A smaller G‐ratio refers to a higher myelin thickness relative to axon diameter. The mean axonal diameter exhibited no difference between genotypes (wild type: 1.74 ± 0.06 μm; *Elovl5* ^*−/−*^ mice: 1.71 ± 0.22 μm; Student's *t*‐test *t*_[5]_ = 0.09, *p* > .05) and also their frequency distribution (Figure 1(e), Mann–Whitney *U*‐test, *p* = .20), while myelin thickness was significantly larger in *Elovl5* ^*−/−*^mice (wild type: 0.66 ± 0.08 μm; *Elovl5* ^*−/−*^ mice: 0.97 ± 0.09 μm; Unpaired Student's *t*‐test *t*_[7]_ = 2.62, *p* < .05, Figure 1(f)). To investigate the ultrastructural cause of the increased myelin thickness, the period between myelin layers was analyzed. *Elovl5* ^*−/−*^ sciatic nerves showed expanded myelin periodicity relative to wild type nerves (wild type: 19.35 ± 0.66 nm; *Elovl5* ^*−/−*^ mice: 21.67 ± 0.26 nm; Student's *t*‐test *t*_[4]_ = 3.21, *p* < .05, Figure 1(b)).

### 3.2 Increased nodal gap and paranode length in sciatic nerves of *Elovl5* ^*−/−*^ mice

**FIGURE 2:**
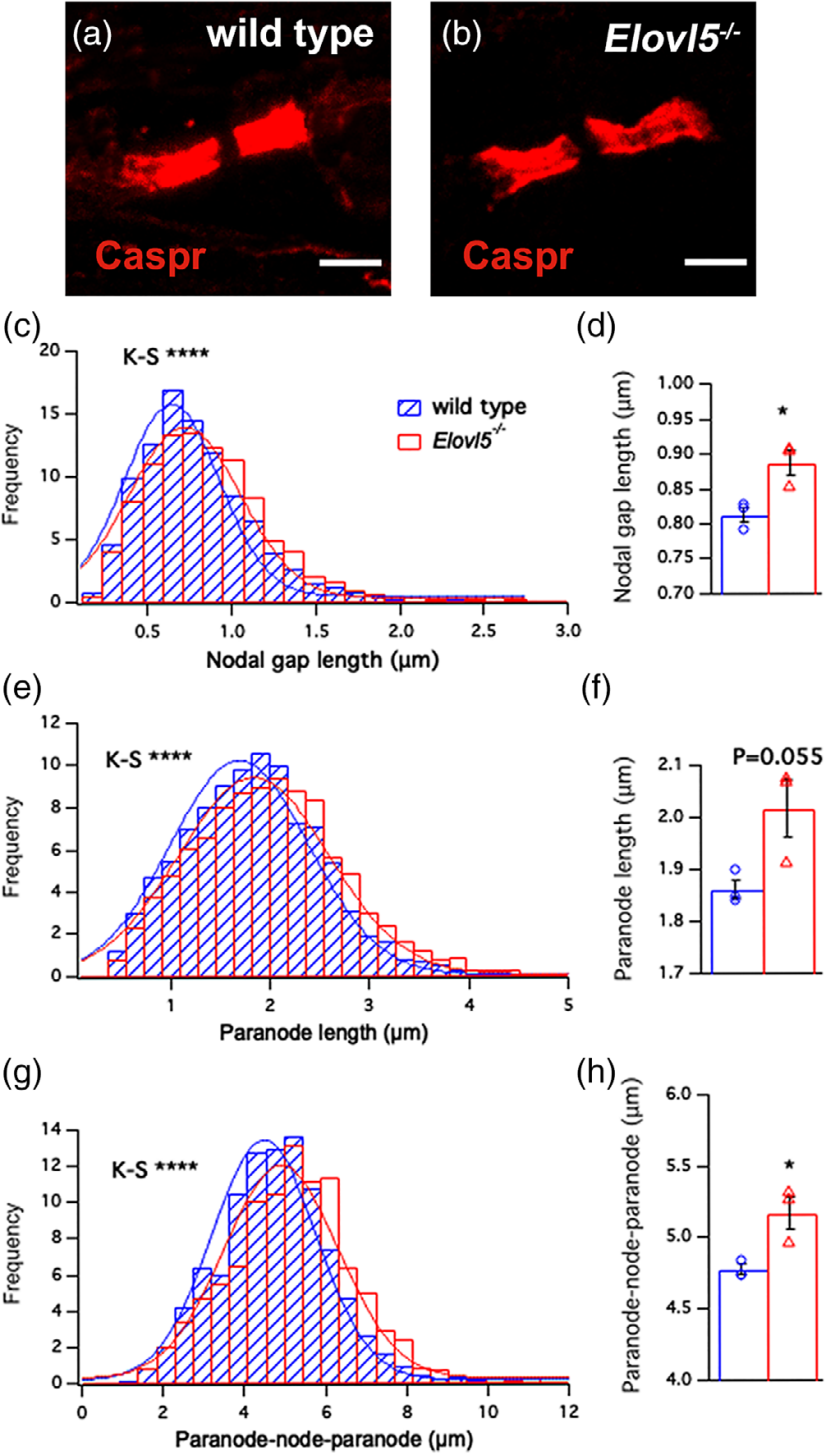
Increased length of the node/paranode complexes of sciatic nerves of *Elovl5* ^−/−^ mice. Confocal images of a single teased sciatic nerve from (a) wild type (n = 3) and (b) *Elovl5* ^−/−^ mice (n = 3) showing paranodes labeled for Caspr (red). (c) Histogram distribution of nodal gap length (*p* < .001, Kolmogorov–Smirnov test) and (d) mean ± SEM of the node lengths for wild type (blue) and *Elovl5* ^−/−^ mice (red) (Student's *t*‐test, *p* < .05). (e) Histogram distribution of paranodal length (*p* < .001, Kolmogorov–Smirnov test) and (f) mean ± SEM of paranodal length (Student's *t*‐test, *p* < .05). (g) Histogram distribution of node/paranode length (*p* < .001, Kolmogorov–Smirnov test) and (h) mean ± SEM of node/paranode length (Student's *t*‐test, *p* < .05). *p < .05; ***p < .001

We next addressed the possibility that the decompaction of myelin in nerves of *Elovl5* ^*−/−*^ mice was associated with alterations of axonal domain organization. By means of confocal microscopy performed on teased sciatic nerve fibers, we analyzed the node/paranode complexes (where the nodal gap is the space flanked by two Caspr positive paranodes) in *Elovl5* ^*−/−*^ mice and wild type littermates (Figure 2(a,b)). Interestingly, we found that the distribution of measurements of the nodal gap length in *Elovl5* ^*−/−*^ mice was significantly shifted to the right (Kolmogorov–Smirnov test, *D* = 0.10, *p* < .001, Figure 2(c)) with a mean nodal gap length significantly higher (wild type: 0.81 ± 0.01 μm; *Elovl5* ^*−/−*^ mice: 0.87 ± 0.02 μm; Student's *t*‐test *t*_[4]_ = 3.43, *p* < .05, Figure 2(d)). The increase in the nodal gap length was also accompanied by an increase of the average Caspr domain length (Kolmogorov–Smirnov test, *D* = 0.10, *p* < .001, Figure 2(e) **)** with a tendency to higher values of the mean nodal gap (wild type: 1.86 ± 0.02 μm; *Elovl5* ^*−/−*^ mice: 2.02 ± 0.05 μm; Student's *t*‐test *t*_[4]_ = 2.69, *p* = .055, Figure 2(f)). The increase of the nodal gap and the mean paranode length is reflected in a stretched structure of the complex node‐paranode in *Elovl5* ^*−/−*^ mice (Kolmogorov–Smirnov test, *D* = 0.14, *p* < .001, Figure 2(g), wild type: 4.78 ± 0.03 μm; *Elovl5* ^*−/−*^ mice: 5.17 ± 0.11 μm; Student's *t*‐test *t*_[4]_ = 3.43, *p* < .05, Figure 2(h)) which might affect the action potential conduction along myelinated axons.

### 3.3 Reduced action potential propagation in peripheral axons of *Elovl5* ^*−/−*^ mice

**FIGURE 3:**
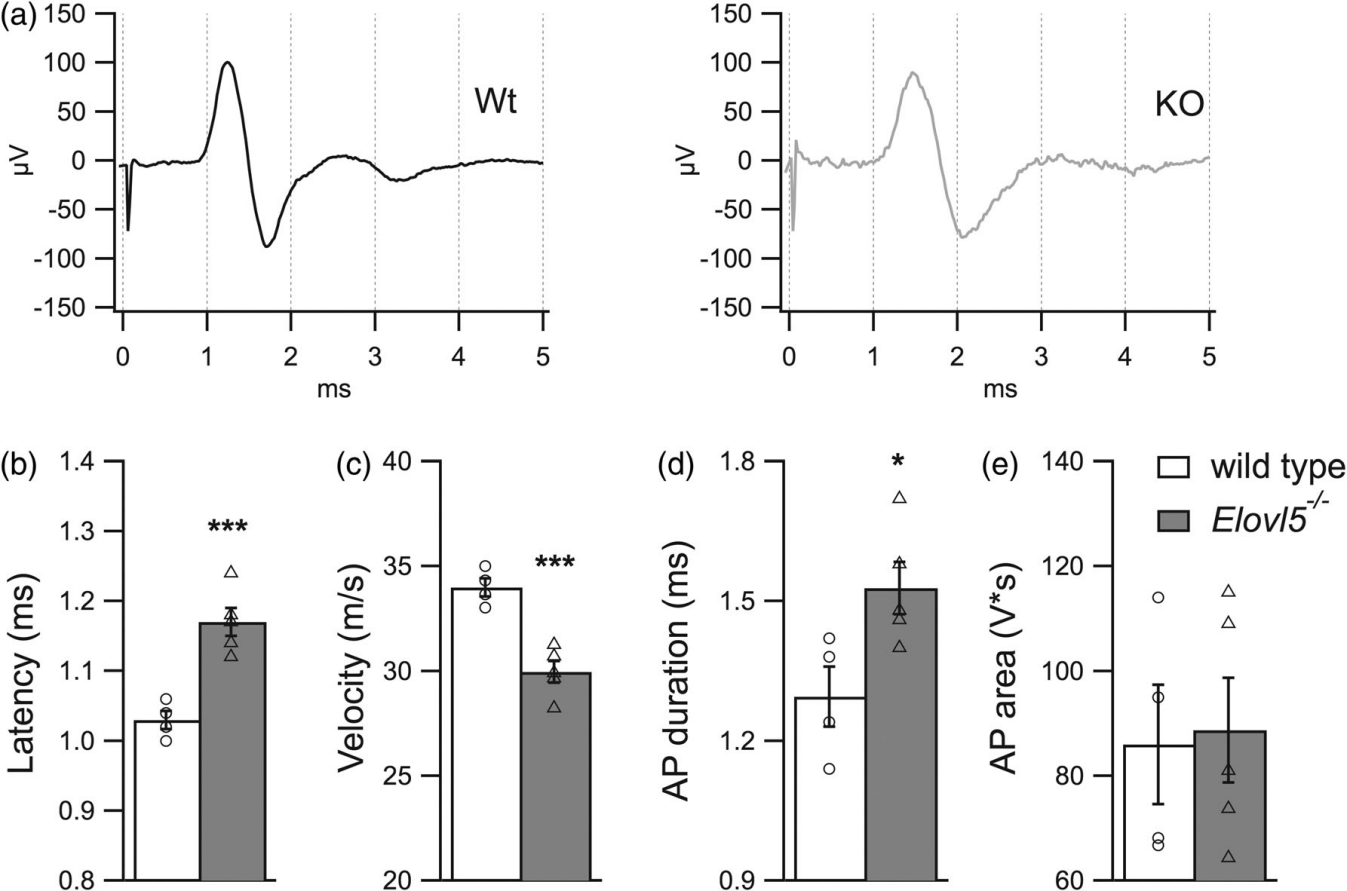
Reduced action potential propagation in peripheral axons of *Elovl5* ^*−/−*^ mice. (a) Representative traces of action potentials evoked by stimulation of the tail nerve for wild type (black) and *Elovl5* ^*−/−*^ mice (light gray). (b–e) Bar graphs representing mean values of latency, nerve conduction velocity, action potential duration and action potential area respectively (*Elov5* ^*−/−*^ n = 5 mice vs. wild type n = 4 mice). Data are expressed as mean ± SEM and *p*‐values are determined by unpaired Student's *t*‐test. *p < .05; ***p < .001

To study the contribution of Elovl5‐dependent fatty acids on myelin functioning in the peripheral nervous system we performed action potential recordings on the caudal nerve of *Elovl5* ^*−/−*^ (n = 5) and wild type mice (n = 4, Figure 3(a) ). *Elovl5* ^*−/−*^ mice showed a significant increase in the latency of the action potential (AP) relative to their wild type littermates (wild type: 1.03 ± 0.0001 ms, n = 4; *Elovl5* ^*−/−*^ mice: 1.17 ± 0.00002 ms, n = 5; Unpaired Student's *t*‐test *t*_[7]_ = 5.41, *p* < .001, Figure 3(b) ) and a significant decrease of conduction velocity (wild type: 33.40 ± 0.43 m/s; *Elovl5* ^*−/−*^ mice: 29.95 ± 0.52 m/s; Unpaired Student's *t*‐test *t*_[7]_ = 5.83, *p* < .001, Figure 3(c) ). Moreover, the AP duration was significantly longer in *Elovl5* ^*−/−*^ mice (wild type: 1.3 ± 0.06 ms; *Elovl5* ^*−/−*^ mice: 1.53 ± 0.06 ms; Unpaired Student's *t*‐test *t*_[7]_ = 2.74, *p* < .05, Figure 3(d) ). On the other hand, no difference was observed for AP area (wild type: 86 ± 11.4 V*s; *Elovl5* ^*−/−*^ mice: 88.7 ± 10 V*s; Unpaired Student's *t*‐test *t*_[7]_ = 0.18, *p* > .05).

### 3.4 Myelin proteins in sciatic nerve of *Elovl5* ^*−/−*^ mice

**FIGURE 4:**
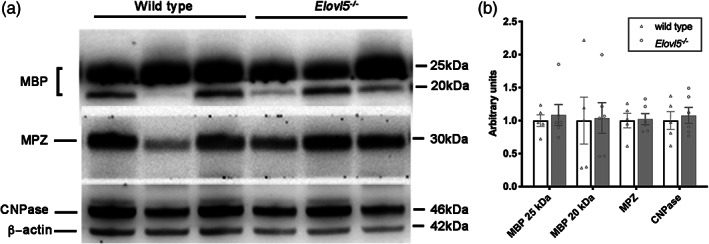
Proteins of myelin. (a) Representative western blots of sciatic nerve extracts from wild type and *Elov5* ^*−/−*^ mice. (b) Densitometric quantification shows comparable levels of MBP, MPZ, and CNPase proteins in the sciatic nerve extracts of *Elov5* ^*−/−*^ mice compared to their control littermates (wild type n = 5; *Elov5* ^*−/−*^ n = 6). β Actin served as loading control

Myelin possesses a peculiar structure that differs from other membranes for the high lipid to protein ratio. Proteins participate in several mechanisms including stabilization of the structure of myelin sheaths or signaling during myelination (Campagnoni & Skoff, 2006). To verify whether the expanded myelin periodicity, in sciatic nerve of *Elovl5* ^*−/−*^ mice, was accompanied by changes in protein expression we performed western blot analysis. We found unchanged levels of, MPZ, MBP, and CNPase proteins in *Elovl5* ^*−/−*^ compared to wild type sciatic nerves (Student's *t*‐test, *p* > .05, Figure 4(a,b)).

### 3.5 Phospholipid profile of *Elovl5* ^*−/−*^ sciatic nerve

Based on the myelin defects described above and considering that Elovl5 is involved in fatty acid elongation, we next sought to determine the profile of phospholipids. The sciatic nerves were extracted from wild type and *Elovl5* ^*−/−*^ mice (n = 5–6 mice/genotype) and the lipidomic profile was resolved. The composition in terms of phospholipid species detected across the two experimental groups was comparable as well as the total amount of phospholipids detected (Figure S1). However, we detected 46 different phospholipids that were significantly affected by the lack of Elovl5 (Table S2). Specifically, 2 lysophosphatidylcholines (lyso PC), a phosphatidylglycerol, a lysophosphatidic acid (LPA), 2 phosphatidic acids (PA), 2 phosphatidylinositols (PI), a phosphatidylserine (PS), a sphingomyelin (SM), 2 ceramides (Cer), 3 sulfatides (Sul), 7 phosphatidylcholines (PCaa) and 13 phosphatidylethanolamines (PEaa) carrying different fatty acids bound to the glycerol moiety by two ester linkages at both *sn‐1* and *sn‐2* position (di‐acyl form, therefore aa means acyl‐acyl), 11 plasmalogens (these molecules are phospholipids characterized by the presence of a vinyl ether linkage at the *sn‐1* position and an ester linkage at the *sn‐2* position of the glycerol moiety; (alkyl‐acyl form, therefore ae means alkyl‐acyl) (Table S2). The most common plasmalogens in mammals carry either ethanolamine (plasmenylethalomines) or choline (plasmenylcholines) as head group.

**FIGURE 5:**
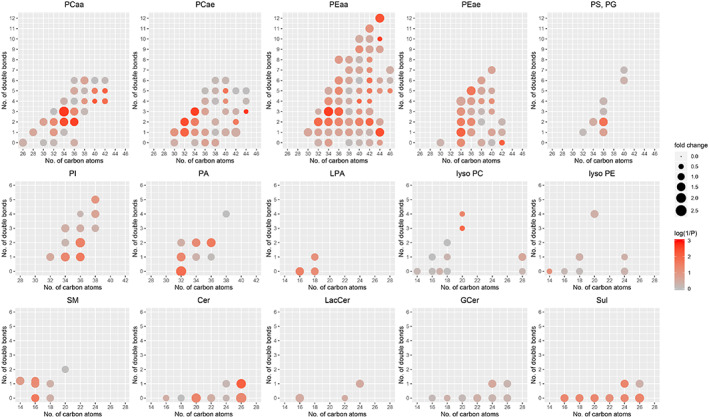
Altered phospholipid profile of *Elovl5* ^*−/−*^ sciatic nerve. Fold change of the main classes of phospholipids in *Elov5* ^*−/−*^ sciatic nerve relative to wild type. The fold change is represented by the size of the circles (see scale legend on the right). The color (from gray to red) represents the statistical significance level (1/*p*) with full red corresponding to *p* ≤ .001 (see color legend on the right). Note that the significant increases concern phospholipids with 2 acyl chains with 3 or <3 unsaturated bonds and with up to 36 carbon atoms. The significantly decreased phospholipids with 2 acyl chains have >3 unsaturated bonds and >36 carbon atoms. PCaa, phosphatidylcholines; PCae, plasmenylcholines; PEaa, phosphatidylethanolamines; PEae, plasmenylethalomines; PS, phosphatidylserines; PG, phosphatidylglycerols; PI, phosphatidylinositols; PA, phosphatidic acids; LPA, lysophosphatidic acids; lyso PC, lysophosphatidylcholines; lyso PE, lysophosphatidylethanolamines; SM, sphingomyelins and sphingomyelins(OH) (the latter are plotted slightly upward shifted); Cer, ceramides; LacCer, lactosylceramides; GCer, glucosylceramides; Sul, sulfatides

Among phospholipids with 2 acyl chains, we found that *Elovl5* ^*−/−*^ peripheral nerves showed increased levels of those with 3 or <3 unsaturated bonds and with up to 36 carbon atoms (Figure 5). The only exceptions are PEaa with lower saturation (44:1 and 42:2), PEaa 44:12 and PEae 36:5. On the other hand, significantly decreased phospholipids with 2 acyl chains had >36 carbon atoms and >3 unsaturated bonds. This result agrees with the reduction of lysoPCs (which have a single acyl chain) with 20 carbon atoms and 3 or 4 unsaturated bonds. We detected mainly saturated or monounsaturated sphingomyelins, ceramides and sulfatides, with a few instances of increased expression Figure 5).

Together, these data demonstrate that despite a comparable composition in terms of phospholipid families between wild type and *Elovl5* ^*−/−*^ sciatic nerves, the lack of Elovl5 negatively impacts some phospholipids that contribute to myelin compaction.

## 4 DISCUSSION

In the current study, we investigated the consequences of Elovl5 deficiency in peripheral nerves. We exploited *Elovl5* ^*−/−*^ mice and show that the lack of Elovl5 enzymatic activity in mice leads to biochemical and structural changes in myelin, which have functional consequences in the velocity of action potential conduction along axons.

Our lipidomic analysis, in line with the lack of Elovl5, revealed an accumulation of phospholipids consisting of 16 or 18 carbons fatty acids (Elovl5 substrates) with at most two or three unsaturations. On the other hand, *Elovl5* ^*−/−*^ fibers showed a strong reduction of phospholipids carrying the majority of fatty acids composed of at least 20 carbons with multiple unsaturations (Elovl5 products). More specifically, the strong alteration of the plasmalogens observed in *Elovl5* ^*−/−*^ mice is in line with the finding of a less compacted myelin. Indeed, plasmalogens represent a substantial part of phospholipids and are reported to protect myelin structure from oxidative stress, so that changes in their quantity can influence myelin‐packing properties (Luoma et al., 2015).

Noteworthy, the most prominent effect of the lack of Elovl5 on the lipidic profile is a reduced ratio between polyunsaturated versus saturated and monounsaturated fatty acids. Interestingly, impaired PUFA levels, in the liver, are shown to increase the activity of the sterol regulatory element‐binding protein (Srebp‐1c) that pushes the expression of different lipogenic genes implicated in the monounsaturated and saturated fatty acid synthesis in *Elovl5* ^*−/−*^ mice (Moon et al., 2009). However, our analysis in the sciatic nerve did not reveal changes in the Srebp‐1c and proteins involved in synthesis of phospholipids (Figure S2). Actually, we found that Schwann cells themselves express Elovl5 indicating local PUFAs synthesis (Figure S3). This finding raises the question of the role of intrinsic synthesis relative to the uptake of preformed lipids from the bloodstream.

From a functional point of view, saturated fatty acids lead to stronger lipid‐lipid interactions and make membranes more rigid and tightly packed, while PUFAs fluidize membranes (Harayama & Riezman, 2018; Sezgin et al., 2017; van Meer et al., 2008). Moreover, the lack of Elovl5 revealed an important accumulation of sphingolipids, which are structural lipids highly enriched in nervous cells, and beyond their role in the architecture of membranes also participate in different cellular pathways (Venkataraman & Futerman, 2000). A 30% increase of sphingomyelin and galactosylceramide in lipid bilayer models is sufficient to cause an increase of the membrane stiffness and reduced flexibility (Saeedimasine et al., 2019). Sphingomyelin rich bilayers tend to form hydrogen bonding together (Niemelä et al., 2004), while galactosylceramides tend to pack together via sugar‐sugar bonding and to make strong interactions with phospholipids, thus causing thickening of the membrane (Saeedimasine et al., 2019). Indeed, the accumulation of sphingolipids is the main feature of lipid storage diseases (Sural‐Fehr & Bongarzone, 2016; Zheng et al., 2006), which are associated with aberrant myelination and peripheral neuropathy (Bagel et al., 2013; Higashi et al., 1995; Ramakrishnan et al., 2007).

Even though there are several studies demonstrating that some specific lipids influence structural stability of myelin, there is insufficient information on how changes of the whole lipid composition impact the structure and function of myelin, mainly due to technical difficulties.

Advances in lipidomic investigation highlight the fact that a deranged lipid homeostasis accompanied by myelin defects and axonal conduction deficits are common features for different neurodegenerative diseases (Harel et al., 2018; Horibata et al., 2018; Karsai et al., 2019; Kutkowska‐Kaźmierczak et al., 2018; Pujol‐Lereis, 2019; Vaz et al., 2019).

In line with alterations of phospholipid profile, in *Elovl5* ^*−/−*^ nerves, myelin thickness was increased, and the layer periodicity was enlarged. This corresponds to a deficit in myelin compactness, which is required to provide better electrical insulation, reduction of membrane capacitance and faster action potential conduction (Schmidt & Knösche, 2019). Interestingly, an increase in myelin periodicity is reported in mice with defects in fatty acid synthesis (Cermenati et al., 2015) and in mice with deficiency of plasmalogens (da Silva et al., 2014).

The node/paranode structure is strongly dependent on the lipid composition (Thaxton and Bhat, 2009). Accordingly, the nodal gap and the paranode length are increased in *Elovl5* ^*−/−*^ nerves, suggesting a role of Elovl5‐dependent phospholipids in the maintenance of integrity of nodes and paranodes. It is well reported that the lack of sulfatides causes nodal and paranodal junction abnormalities in mice (Ishibashi et al., 2002; Marcus et al., 2006; Takano et al., 2012).

Unsurprisingly, in *Elovl5* ^*−/−*^ mice, such deficits in myelin compactness and length of nodes and paranodes were associated with a slower conduction velocity of action potentials. Given the importance of myelin thickness, node and paranode length in influencing the velocity of action potentials (Schmidt & Knösche, 2019), it is not surprising to find that the alteration of even one of these parameters will cause deficits in the action potential conduction along axons (Arancibia‐Cárcamo et al., 2017; Li, 2015).

The high amount of lipids in myelin renders them important players in determining structural integrity of myelin therefore influencing the conduction of action potentials. In summary, our findings strengthen the notion that the Elovl5 enzyme is necessary for a correct maintenance of the homeostasis of fatty acids in peripheral myelin, which is crucial to assure the correct biophysical properties of the membrane.

## CONFLICT OF INTEREST

The authors declare no conflict of interest.

## Supporting information

**FIGURE S1.** Total content of sciatic nerve myelin phospholipids. Representation of the percentages of the different phospholipid species detected in sciatic nerve of wild type and Elovl5−/− mice

**FIGURE S2.** Gene expression analysis performed in the sciatic nerve obtained from wild type (n = 5 mice) and Elovl5−/− mice (n = 5). The analysis showed no significant difference between *Elovl5* ^*−/−*^ and wild type littermates in the level of transcription factors Srebp1c and Srebp2, and some targets of the Srebp pathway like Elovl6 and Scd2 (*p* > .05, Unpaired Student's *t*‐test)

**FIGURE S3.** Elovl5 expression by RT4‐D6P2T cell line and gene expression of Elovl5 in sciatic nerves. (a) Representative images showing RT4‐D6P2T cells stained with Elovl5 antibody (red) and DAPI (blue). (b) Gene expression analysis of Elovl5 in sciatic nerves of wild type mice. Relative gene expression was calculated by the normalized comparative cycle threshold (Ct) method 2^−ΔCt^

**TABLE S1.** List of the applied biosystems' TaqMan gene expression assays or Roche diagnostics's combinations of primers and UPL probe used in the present study

**TABLE S2.** Quantification of sciatic nerve phospholipids

**DATA S1.** Supporting Information.

## Data Availability

The data that support the findings of this study are available upon reasonable request.
