# Supplementary figures and images for: Elovl5 is required for proper action potential conduction along peripheral myelinated fibers
Source: Glia. 2021 Jun 17;69(10):2419–28. doi: 10.1002/glia.24048 (PMC8453547; doi:10.1002/glia.24048)

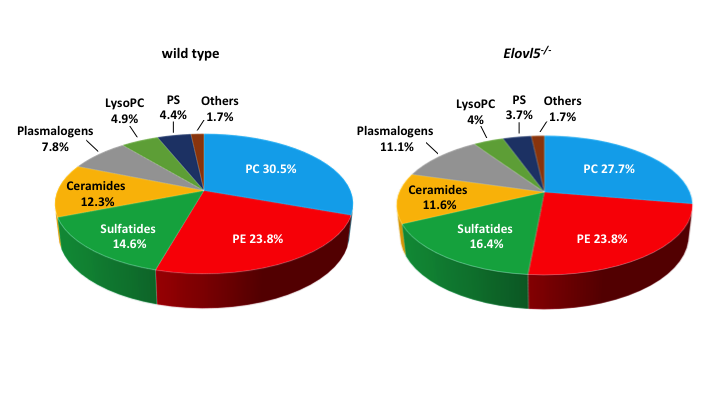

Supplement: Supplementary file 1 — FIGURE S1. Total content of sciatic nerve myelin phospholipids. Representation of the percentages of the different phospholipid species detected in sciatic nerve of wild type and Elovl5−/− mice [file GLIA-69-2419-s004.tif]

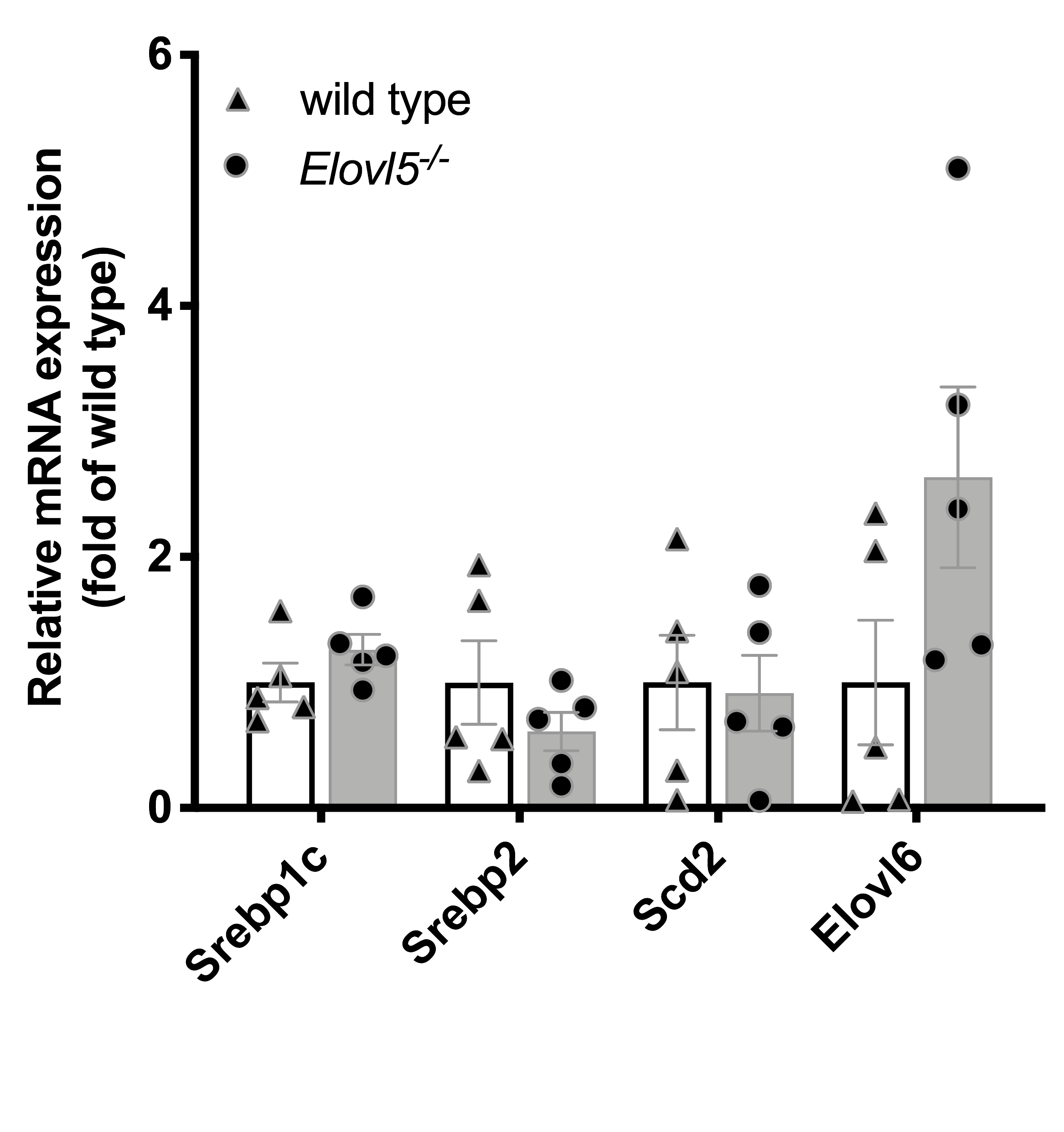

Supplement: Supplementary file 2 — FIGURE S2. Gene expression analysis performed in the sciatic nerve obtained from wild type (n = 5 mice) and Elovl5−/− mice (n = 5). The analysis showed no significant difference between Elovl5 −/− and wild type littermates in the level of transcription factors Srebp1c and Srebp2, and some targets of the Srebp pathway like Elovl6 and Scd2 (p > .05, Unpaired Student's t‐test) [file GLIA-69-2419-s003.tif]

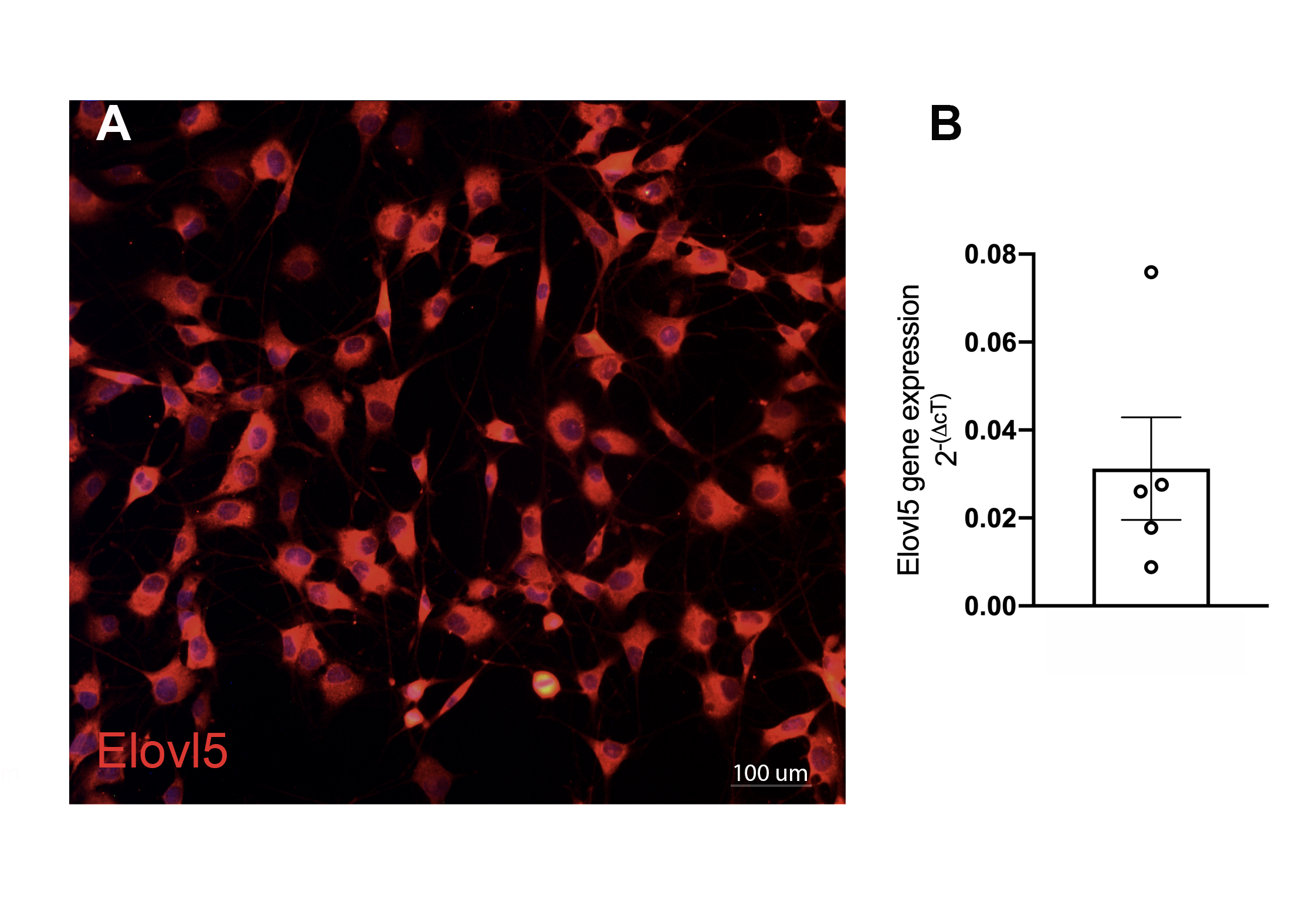

Supplement: Supplementary file 3 — FIGURE S3. Elovl5 expression by RT4‐D6P2T cell line and gene expression of Elovl5 in sciatic nerves. (a) Representative images showing RT4‐D6P2T cells stained with Elovl5 antibody (red) and DAPI (blue). (b) Gene expression analysis of Elovl5 in sciatic nerves of wild type mice. Relative gene expression was calculated by the normalized comparative cycle threshold (Ct) method 2−ΔCt [file GLIA-69-2419-s006.tif]
