# Supplementary material for: Elovl5 is required for proper action potential conduction along peripheral myelinated fibers
Source: Glia. 2021 Jun 17;69(10):2419–28. doi: 10.1002/glia.24048 (PMC8453547; doi:10.1002/glia.24048)
Supplement: Supplementary file 4 — TABLE S1. List of the applied biosystems' TaqMan gene expression assays or Roche diagnostics's combinations of primers and UPL probe used in the present study [file GLIA-69-2419-s001.docx]

**Supplementary Table 1.** List of the Applied Biosystems’ TaqMan gene expression assays or Roche Diagnostics’s combinations of primers and UPL probe used in the present study.

| **Target gene** | **Applied Biosystems’ TaqMan** | **Roche Diagnostics’s**  **Primers + UPL probe** |
| --- | --- | --- |
| Glyceraldehyde-3-phosphate dehydrogenase (GAPDH) | Mm99999915_g1 |  |
| ELOngase of Very Long chain fatty acids type 5 (Elovl5) |  | FW: gtcctccatcccgtccat  RV: gctgcccttgagtgatgtact  Probe #31 |
| ELOngase of Very Long chain fatty acids type 6 (Elovl6) |  | FW: cagcaaagcacccgaacta  RV: aggagcacagtgatgtggtg  Probe #4 |
| Sterol regulatory element-binding factor 1 (Srebp1c) |  | FW: acaagattgtggagctcaaagac  RV: tgcgcaagacagcagattta  Probe #77 |
| Sterol regulatory element-binding factor 2 (Srebp2) |  | FW: ccctattccattgactctgagc  RV: gagtccggttcatccttgac  Probe #42 |
| Stearoyl-CoA-desaturase 2 (Scd2) |  | FW: tggtttccatgggagctg  RV: ttgatgtgccagcggtact  Probe #53 |
