# Supplementary material for: Elovl5 is required for proper action potential conduction along peripheral myelinated fibers
Source: Glia. 2021 Jun 17;69(10):2419–28. doi: 10.1002/glia.24048 (PMC8453547; doi:10.1002/glia.24048)
Supplement: Supplementary file 5 — TABLE S2. Quantification of sciatic nerve phospholipids [file GLIA-69-2419-s005.docx]

**Supplementary table 2.** Quantification of sciatic nerve phospholipids

| Phospholipid name | wild type  (ng/mg) | *Elovl5^-/-^*  (ng/mg) | t, df | P value |
| --- | --- | --- | --- | --- |
| lysoPC C20:4 | 0.269±0.045 | 0.149±0.013 | 2.366, 9 | 0.0422 |
| lysoPC C20:3 | 0.031±0.005 | 0.016±0.002 | 3.073, 10 | 0.0118 |
| PC aa C34:3 | 0.168±0.013 | 0.337±0.039 | 4.078, 10 | 0.0022 |
| PC aa C34:2 | 4.986±0.480 | 7.232±0.365 | 3.725, 10 | 0.0039 |
| PC aa C36:2 | 3.937±0.322 | 5.479±0.223 | 3.933, 10 | 0.0028 |
| PC aa C40:5 | 0.160±0.014 | 0.113±0.013 | 2.480, 10 | 0.0325 |
| PC aa C40:4 | 0.415±0.030 | 0.180±0.060 | 3.502, 10 | 0.0057 |
| PC aa C42:5 | 0.072±0.005 | 0.039±0.009 | 3.231, 10 | 0.0090 |
| PC aa C42:4 | 0.195±0.013 | 0.124±.0.020 | 2.982, 10 | 0.0138 |
| PC ae C32:2 | 0.035±0.004 | 0.050±0.001 | 3.754, 10 | 0.0038 |
| PC ae C32:1 | 0.076±0.007 | 0.117±0.010 | 3.227, 10 | 0.0091 |
| PC ae C34:3 | 0.046±0.004 | 0.067±0.003 | 3.972, 10 | 0.0026 |
| PC ae C34:2 | 0.669±0.041 | 0.881±0.075 | 2.580, 9 | 0.0297 |
| PC ae C40:5 | 0.048±0.004 | 0.027±0.006 | 2.782, 10 | 0.0194 |
| PC ae C42:2 | 0.013±0.001 | 0.008±0.001 | 2.688, 9 | 0.0249 |
| PC ae C44:3 | 0.007±0.001 | 0.003±0.001 | 4.125, 10 | 0.0021 |
| PE aa 32:2 | 0.058±0.007 | 0.088±0.007 | 3.049, 10 | 0.0123 |
| PE aa 34:3 | 0.222±0.025 | 0.389±0.034 | 3.984, 10 | 0.0026 |
| PE aa 34:2 | \| 1.303±0.138 \|  \| \| --- \| --- \| | 1.831±0.123 | 2.862, 10 | 0.0169 |
| PE aa 36:3 | \| 1.626±0.183 \|  \| \| --- \| --- \| | 2.499±0.192 | 3.285, 10 | 0.0082 |
| PE aa 36:2 | \| 5.340±0.668 \|  \| \| --- \| --- \| | 7.875±0.960 | 2.252, 10 | 0.0480 |
| PE aa 40:4 | \| 0.6457±0.095 \|  \| \| --- \| --- \| | 0.272±0.073 | 3.108, 10 | 0.0111 |
| PE aa 40:2 | \| 0.465±0.052 \|  \| \| --- \| --- \| | 0.648±0.055 | 2.418, 10 | 0.0362 |
| PE aa 42:6 | \| 0.057±0.006 \|  \| \| --- \| --- \| | 0.037±0.003 | 2.850, 10 | 0.0173 |
| PE aa 42:5 | \| 0.113±0.015 \|  \| \| --- \| --- \| | 0.063±0.012 | 2.693, 10 | 0.0226 |
| PE aa 42:4 | \| 0.124±0.022 \|  \| \| --- \| --- \| | 0.054±0.014 | 2.741, 10 | 0.0208 |
| PE aa 42:2 | \| 0.793±0.043 \|  \| \| --- \| --- \| | 0.980±0.041 | 3.141, 10 | 0.0105 |
| PE aa 44:12 | \| 0.141±0.013 \|  \| \| --- \| --- \| | 0.226±0.020 | 3.590, 10 | 0.0049 |
| PE aa 44:10 | \| 0.282±0.026 \|  \| \| --- \| --- \| | 0.110±0.034 | 3.999, 10 | 0.0025 |
| PE aa 44:1 | \| 0.009±0.001 \|  \| \| --- \| --- \| | 0.014±0.001 | 3.661, 10 | 0.0044 |
| PE aa 46:5 | \| 0.005±0.001 \|  \| \| --- \| --- \| | 0.003±0.001 | 2.401, 10 | 0.0373 |
| PE ae 34:2 | \| 0.020±0.002 \| \| --- \| | 0.028±0.002 | 2.568, 10 | 0.0280 |
| PE ae 34:1 | \| 0.065±0.009 \|  \| \| --- \| --- \| | 0.105±0.009 | 3.230, 10 | 0.0090 |
| PE ae 36:5 | \| 0.015±0.002 \|  \| \| --- \| --- \| | 0.025±0.003 | 2.766, 10 | 0.0199 |
| PE ae 42:0 | \| 0.112±0.011 \|  \| \| --- \| --- \| | 0.065±0.008 | 3.310, 10 | 0.0079 |
| SM 16:0 | \| 0.092±0.010 \|  \| \| --- \| --- \| | 0.120±0.004 | 2.632, 10 | 0.0251 |
| Cer-d18:1/20:0 | 0.001±0.0001 | 0.002±0.0003 | 2.310, 10 | 0.0435 |
| Cer-d18:1/26:1 | \| 0.020±0.003 \|  \| \| --- \| --- \| | 0.036±0.004 | 3.284, 10 | 0.0082 |
| PG 36:3 | \| 0.038±0.003 \|  \| \| --- \| --- \| | 0.056±0.006 | 2.828, 10 | 0.0179 |
| PA 32:0 | \| 0.030±0.008 \|  \| \| --- \| --- \| | 0.064±0.008 | 2.945, 10 | 0.0147 |
| PA 36:2 | \| 0.031±0.004 \|  \| \| --- \| --- \| | 0.051±0.006 | 2.622, 10 | 0.0255 |
| LPA 16:0 | \| 0.082±0.004 \|  \| \| --- \| --- \| | 0.117±0.007 | 4.247, 10 | 0.0017 |
| PI 36:2 | \| 0.014±0.002 \|  \| \| --- \| --- \| | 0.027±0.005 | 2.382, 10 | 0.0385 |
| PI 36:1 | \| 0.025±0.002 \|  \| \| --- \| --- \| | 0.038±0.005 | 2.592, 10 | 0.0269 |
| Sul-d18:1/24:1 | \| 11.350±0.896 \|  \| \| --- \| --- \| | 14.650±1.114 | 2.309, 10 | 0.0436 |
| Sul-d18:1/26:0 | \| 0.136±0.030 \|  \| \| --- \| --- \| | 0.223±0.022 | 2.332, 10 | 0.0419 |

LysoPC, lyso-phosphatidylcholine; PC, phosphatidylcholine; aa, acyl-acyl; ae, acyl-alkyl; PE, phosphatidylethanolamine; SM, sphingomyelin; Cer, ceramides; PG, phosphatidylglycerol; PA, phosphatidic acid; LPA, lysophosphatidic acid; PI, phosphatidylinositol; Sul, sulfatide.
