# Supplementary material for: Elovl5 is required for proper action potential conduction along peripheral myelinated fibers
Source: Glia. 2021 Jun 17;69(10):2419–28. doi: 10.1002/glia.24048 (PMC8453547; doi:10.1002/glia.24048)
Supplement: Supplementary file 6 — DATA S1. Supporting Information. [file GLIA-69-2419-s002.docx]

**Supplementary figure legends**

**Suppl. Fig. 1. Total content of sciatic nerve myelin phospholipids.** Representation of the percentages of the different phospholipid species detected in sciatic nerve of wild type and Elovl5-/- mice.

**Suppl. Fig. 2. Gene expression analysis performed in the sciatic nerve obtained from wild type (n = 5 mice) and *Elovl5^-/-^* mice (n = 5).** The analysis showed no significant difference between *Elovl5^-/-^* and wild type littermates in the level of transcription factors Srebp1c and Srebp2, and some targets of the Srebp pathway like Elovl6 and Scd2 (P > 0.05, Unpaired Student’s t-test).

**Suppl. Fig. 3. Elovl5 expression by RT4-D6P2T cell line and gene expression of Elovl5 in sciatic nerves. (A)** Representative images showing RT4-D6P2T cells stained with Elovl5 antibody (red) and DAPI (blue). **(B)** Gene expression analysis of Elovl5 in sciatic nerves of wild type mice. Relative gene expression was calculated by the normalized comparative cycle threshold (Ct) method 2^−ΔCt^

**Supplementary experimental procedures**

**Real time RT-PCR**

12-months-old Elovl5-/- (n=5) and wild type littermates (n=5) mice were euthanized by inhalation of isoflurane. Sciatic nerves were manually dissected and rapidly frozen in 2-methylbutane in dry ice. Total RNA from sciatic nerves was isolated by extraction with the Pure Link RNA Mini Kit (12183018A, Thermo Fisher Scientific). The RNA was reverse-transcribed to complementary DNA (cDNA) at a final concentration of 10 ng/μl using the High Capacity Kit (4368814, Thermos Fisher Scientific). The cDNA obtained from sciatic nerve was pre-amplified with the TaqMan PreAmp Master Mix (4488593, Thermos Fisher Scientific). Quantitative Real Time reverse transcriptase PCR (RT-PCR) was performed either with Applied Biosystems’ TaqMan gene expression assays (Thermos Fisher Scientific) or by combining the Real Time Ready Universal Probe Library (UPL, Roche Diagnostics, Monza, Italy) with the primers listed in Suppl. Table 1. Transcriptional expression was normalized using glyceraldehyde-3-phosphate dehydrogenase (GAPDH) as reference gene. Expression levels of target genes were calculated by the normalized comparative cycle threshold (Ct) method (2-ΔCt).

**Immunohistochemistry on immortalized rat Schwann cells**

Immortalized rat Schwann cells RT4-D6P2T (ATCC, Manassas, VA, USA) were cultured in Dulbecco’s Modified Eagle Medium (DMEM) (D5796, Sigma Aldrich, MI, USA) supplemented with 10% (v/v) heat-inactivated fetal bovine serum (FBS) (F2442, Sigma Aldrich, MI, USA), 100 U/mL of penicillin, and 100 μg/mL of streptomycin (P4333, Sigma Aldrich, MI, USA). For immunofluorescence staining RT4-D6P2T were cultured on glass cover slides in 12-well cell culture dishes. Cells were fixed with 4% paraformaldehyde in 0.12 M phosphate buffer, pH 7.2–7.4 for 10 min, washed three times with PBS and than incubated with the primary antibody anti-Elovl5 (1:300, Novus Biologicals) overnight at room temperature. The next day after washing with PBS three times, the cells were incubated 1 h at room temperature with secondary antibody donkey anti-rabbit Alexa Fluor 555 (1:500, Invitrogen). After processing, sections were mounted on microscope slides with Pro Long Gold Antifade Reagent with DAPI (P36935, Molecular Probes, Thermo Fisher Scientific, MA, USA).
